# The sensitivity of marine N_2_ fixation to dissolved inorganic nitrogen

**DOI:** 10.3389/fmicb.2012.00374

**Published:** 2012-10-19

**Authors:** Angela N. Knapp

**Affiliations:** Rosenstiel School of Marine and Atmospheric Sciences, University of MiamiMiami, FL, USA

**Keywords:** N2 fixation, diazotroph, inhibition, sensitivity, nitrate, ammonium

## Abstract

The dominant process adding nitrogen (N) to the ocean, di-nitrogen (N_2_) fixation, is mediated by prokaryotes (diazotrophs) sensitive to a variety of environmental factors. In particular, it is often assumed that consequential rates of marine N_2_ fixation do not occur where concentrations of nitrate (NO^−^_3_) and/or ammonium (NH^+^_4_) exceed 1μM because of the additional energetic cost associated with assimilating N_2_ gas relative to NO^−^_3_ or NH^+^_4_. However, an examination of culturing studies and *in situ* N_2_ fixation rate measurements from marine euphotic, mesopelagic, and benthic environments indicates that while elevated concentrations of NO^−^_3_ and/or NH^+^_4_ can depress N_2_ fixation rates, the process can continue at substantial rates in the presence of as much as 30μM NO^−^_3_ and/or 200μM NH^+^_4_. These findings challenge expectations of the degree to which inorganic N inhibits this process. The high rates of N_2_ fixation measured in some benthic environments suggest that certain benthic diazotrophs may be less sensitive to prolonged exposure to NO^−^_3_ and/or NH^+^_4_ than cyanobacterial diazotrophs. Additionally, recent work indicates that cyanobacterial diazotrophs may have mechanisms for mitigating NO^−^_3_ inhibition of N_2_ fixation. In particular, it has been recently shown that increasing phosphorus (P) availability increases diazotroph abundance, thus compensating for lower per-cell rates of N_2_ fixation that result from NO^−^_3_ inhibition. Consequently, low ambient surface ocean N:P ratios such as those generated by the increasing rates of N loss thought to occur during the last glacial to interglacial transition may create conditions favorable for N_2_ fixation and thus help to stabilize the marine N inventory on relevant time scales. These findings suggest that restricting measurements of marine N_2_ fixation to oligotrophic surface waters may underestimate global rates of this process and contribute to uncertainties in the marine N budget.

## Introduction

Phytoplankton growing in the sunlit surface ocean (euphotic zone) produce organic matter via photosynthesis at a rate of ~50PgC year^−1^ (Westberry et al., 2008), and thus play an important role in the global carbon (C) cycle. However, phytoplankton growth in the euphotic zone is commonly limited by the availability of nutrients such as nitrogen (N); consequently, the processes that add and remove N to and from the ocean, respectively, influence the C cycle and thus climate. Unlike the physical processes that supply other biologically necessary elements like phosphorus (P) and iron (Fe) to the ocean (such as atmospheric deposition and fluvial inputs), the dominant process adding N to the ocean, di-nitrogen (N_2_) fixation, is unique in that it is biologically mediated. While N_2_ gas is unavailable to most organisms, certain groups of prokaryotes known as diazotrophs have the enzymatic ability to reduce dissolved N_2_ to ammonium (NH^+^_4_) and assimilate it into their biomass. The ultimate fate of N in diazotrophic biomass is to be cycled into more bio-available forms, including nitrate (NO^−^_3_), that serve as the primary source of assimilative N for non-diazotrophic phytoplankton and bacteria in the ocean. In spite of the fundamental importance of N_2_ fixation in the global C cycle and in supporting the base of the food web, the locations and rates of N_2_ fixation in the ocean are poorly known.

Uncertainty in the rates of marine N_2_ fixation contributes to ambiguity as to whether the modern marine N budget is balanced. Some estimates suggest that rates of N fluxes to the ocean only compensate for one third to one half of the fluxes of N out of the ocean (Codispoti et al., 2001; Codispoti, 2007), while constraints from paleoceanographic and modeling studies indicate that the marine N budget has been balanced to within ~10% over at least the Holocene (Brandes and Devol, 2002; Deutsch et al., 2004). Assuming that the marine N budget is essentially balanced, the discrepancy in N flux estimates requires that rates of marine N_2_ fixation are underestimated and/or that rates of N loss are overestimated. The constraint of an approximately balanced marine N budget also implies that there are feedback mechanisms allowing N_2_ fixation and denitrification, the dominant pathway by which N is lost from the ocean, to respond to each other on relatively short (i.e., ≤1000 years) timescales. Currently, both the size of the fluxes of N to and from the ocean, as well as the nature of potential feedback mechanisms that maintain a balanced marine N budget, remain ill-defined.

While improved knowledge of the marine N cycle requires a multifaceted approach, characterizing the physical and chemical sensitivities of marine diazotrophs to various environmental conditions provides constraints on regions of the ocean that may support diazotrophy. A better understanding of the sensitivities of marine diazotrophs may also reveal mechanisms by which marine N_2_ fixation can respond to changes in rates of marine denitrification. However, our ability to describe the sensitivities of N_2_ fixation depends on the degree to which we understand and have characterized the diversity of marine diazotrophs, an understanding presently limited by the small number of marine diazotrophs isolated for manipulative culture-based experiments.

The majority of marine N_2_ fixation has historically been attributed to the filamentous, non-heterocystous cyanobacteria *Trichodesmium* spp. resident in the warm, stratified, and nutrient-depleted regions of the surface ocean (Carpenter, 1983; Capone et al., 1997, 2005). However, the past decade has seen a number of challenges to the paradigm that N_2_ fixation by *Trichodesmium* spp., especially in the tropical North Atlantic, is the primary source of N to the global ocean. For example, molecular tools have identified novel diazotrophs present in environments with physical and/or chemical characteristics different from their more well-studied counterparts in tropical and subtropical seas (Zehr et al., 2001, 2008; Montoya et al., 2004; Langlois et al., 2008; Moisander et al., 2010; Fernandez et al., 2011). Additionally, indirect evidence such as remote sensing (Westberry et al., 2005; Westberry and Siegel, 2006) and geochemical modeling (Deutsch et al., 2007) describes geographic distributions of N_2_ fixers, including *Trichodesmium* spp., that differs from our expectation of oligotrophic dominance. Finally, a number of both *in situ* and culture-based studies challenge some long-held notions of diazotrophic sensitivities to nutrients, including the degree to which inorganic N inhibits N_2_ fixation. All of these findings raise the possibility that the geographic distribution and sensitivities of marine diazotrophs may be different than previously thought. As recognition of both the breadth of oceanic conditions supportive of diazotrophy and the diversity of marine diazotrophs increases, so too does the possibility that considerable rates of N_2_ fixation occur in environments beyond the surface waters of the oligotrophic gyres. If so, global marine N_2_ fixation rates may be greater than previously estimated.

In spite of an incomplete knowledge of marine diazotroph diversity, environmental and culture-based observations can establish criteria consistent with diazotrophic success. Environmental factors that are known to regulate marine diazotrophy include light (Carpenter et al., 1993; Milligan et al., 2007; Breitbarth et al., 2008), temperature (Chen et al., 1998; Breitbarth et al., 2007; Stal, 2009), oxygen (Robson and Postgate, 1980; Capone and Budin, 1982; Stal and Heyer, 1987), and metal availability (Rueter et al., 1990; Berman-Frank et al., 2001; Kustka et al., 2003; Chappell and Webb, 2010; Saito et al., 2011). Here, the sensitivity of marine diazotrophs to dissolved inorganic N (DIN), in particular NO^−^_3_ and NH^+^_4_, is evaluated, and evidence for the inhibition of N_2_ fixation by DIN in (1) the euphotic zone, (2) the sub-euphotic zone, and (3) benthic marine environments, is reviewed. In particular, the question of whether significant rates of N_2_ fixation can occur when ambient DIN concentrations are significant, i.e., ≥1μM, is examined. The findings of this review are that: (1) reports of substantial rates of N_2_ fixation in euphotic and benthic environments with ≥1μM DIN indicate that elevated DIN does not necessarily preclude large N_2_ fixation fluxes; (2) certain benthic marine diazotrophs may be less sensitive to chronic exposure to elevated concentrations of DIN than diazotrophs in the euphotic zone; (3) while benthic N_2_ fixation is widespread and can occur at significant rates, global estimates are poorly known, likely contributing significant uncertainty to global estimates of marine N_2_ fixation fluxes, and, (4) euphotic zone diazotrophs may respond to changes in ambient N:P ratios, providing a potential mechanism for diazotrophs to respond to changes in denitrification rates and thus to stabilize the marine N inventory. These findings are investigated below.

## Nutrient inhibition of euphotic zone N_2_ fixation

There are three primary lines of evidence for the inhibition of marine N_2_ fixation by inorganic N. The first results from circumstances associated with the origins of marine diazotrophic research. Before molecular tools became widely available, our understanding of marine diazotrophs was largely limited to the study of macroscopic cyanobacteria that could be readily identified and manipulated in field and culture-based studies. The most conspicuous and well-studied marine diazotroph, *Trichodesmium* spp., has predominantly been observed in warm, nutrient depleted regions of the surface ocean (Carpenter, 1983; Capone et al., 1997, 2005). The association of *Trichodesmium* spp. with these environmental characteristics, and the strong bias of studies of marine diazotrophs towards *Trichodesmium* spp., has perhaps unintentionally lead to the expectation that other marine diazotrophs will share the same environmental preferences. The second line of evidence for DIN inhibition of N_2_ fixation comes from calculations showing that it requires ~25% more energy to reduce N_2_ (87 kcal) than NO^−^_3_ (69 kcal) to NH^+^_4_ (Falkowski, 1983). Together with the majority of field observations of diazotrophs from nutrient-depleted tropical surface waters, this additional energetic cost has lead to the assumption that significant rates of N_2_ fixation do not occur in marine environments with ≥1μM DIN.

The third line of evidence for the inhibition of N_2_ fixation by DIN comes from culture studies of marine diazotrophs that test the effects of short-term and/or chronic exposure to NO^−^_3_ and NH^+^_4_ (e.g., Ohki et al., 1991; Mulholland and Capone, 1999; Mulholland et al., 2001; Fu and Bell, 2003; Holl and Montoya, 2005) (Table 1) (the numerous studies of DIN inhibition of fresh water diazotrophs are not reviewed here). These studies have demonstrated that NH^+^_4_ is more effective at inhibiting N_2_ fixation than NO^−^_3_ (Ohki and Fujita, 1982; Ohki et al., 1991; Mulholland et al., 2001), presumably because of the larger energetic cost associated with assimilating N_2_ vs. NH^+^_4_ than with assimilating N_2_ vs. NO^−^_3_. Additionally, these studies have shown that chronic exposure to both NO^−^_3_ and NH^+^_4_ more strongly inhibits N_2_ fixation than does short-term (i.e., less than 24 h) exposure (Ohki et al., 1991; Mulholland et al., 2001; Fu and Bell, 2003; Milligan et al., 2007; Dekaezemacker and Bonnet, 2011; Sandh et al., 2011; Knapp et al., 2012) (Table 1). Supporting these observations of depressed N_2_ fixation rates, physiological changes in *Trichodesmium* have also been documented when cultures are grown with NO^−^_3_ as a source of assimilative N instead of dissolved N_2_ gas. After chronic exposure of *Trichodesmium* cultures to 100 μM NO^−^_3_ (Milligan et al., 2007) demonstrated a down-regulation of Mehler activity relative to cultures grown on N_2_ gas, while (Sandh et al., 2011) found an inhibition of nitrogenase expression and diazocyte development. These effects of NO^−^_3_ on diazotroph physiology suggest that chronic exposure to DIN has a greater impact on N_2_ fixation rates than does short-term exposure.

**Table 1:** **Reports of the inhibition of N_2_ fixation by combined N for marine diazotrophs**.

**Diazotroph**	**Experimental condition**	**Form of combined N**	**Concentration of added combined N**	**Concentration of P**	**Duration of exposure**	**% inhibition of N_**2**_ fixation compared to no-DIN control**	**References**
*Trichodesmium thiebautii*, natural populations	Field manipulations	Chloramphenicol	10 μg mL^−1^	Ambient surface seawater	0–7 h	28% inhibition when added before/early in photoperiod	Capone et al., 1990
*Trichodesmium thiebautii*, natural populations	Field manipulations	Chloramphenicol	10 μg mL^−1^	Ambient surface seawater	0–5 h	Stimulated N_2_ fixation when added in late afternoon	Capone et al., 1990
*Trichodesmium thiebautii*, natural populations	Field manipulations	NH^+^_4_	100 μM	Ambient surface seawater	0–7 h	60% inhibition	Capone et al., 1990
*Trichodesmium* sp. NIBB 1067	Batch culture	NO^−^_3_	2 mM	3.2 μM	7 h	0% inhibition	Ohki et al., 1991
*Trichodesmium* sp. NIBB 1067	Batch culture	NH^+^_4_	20 μM	3.2 μM	7 h	0% inhibition	Ohki et al., 1991
*Trichodesmium* sp. NIBB 1067	Batch culture	urea	500 μM	3.2 μM	3 h	Some inhibition	Ohki et al., 1991
*Trichodesmium* sp. NIBB 1067	Batch culture	NO^−^_3_	2 mM	3.2 μM	Multiple generations	100% inhibition	Ohki et al., 1991
*Trichodesmium* sp. NIBB 1067	Batch culture	NH^+^_4_	20 μM	3.2 μM	Multiple generations	100% inhibition	Ohki et al., 1991
*Trichodesmium* sp. NIBB 1067	Batch culture	urea	500 μM	3.2 μM	Multiple generations	100% inhibition	Ohki et al., 1991
*Trichodesmium* sp. NIBB 1067	Batch culture	NO^−^_3_	150 μM	3.2 μM	Multiple generations	75% inhibition	Mulholland et al., 1999
*Trichodesmium* sp. NIBB 1067	Batch culture	urea	30μM	3.2 μM	Multiple generations	66% inhibition	Mulholland et al., 1999
*Trichodesmium* spp. natural populations	Field manipulations	NH^+^_4_	1 and 5 μM	Ambient surface seawater	23 h	20% inhibition for 1μM and 53% inhibition for 5 μM	Mulholland et al., 2001
*Trichodesmium* spp. natural populations	Field manipulations	NH^+^_4_	10 μM	Ambient surface seawater	0–23 h	28% inhibition after 1–2 h, 99% inhibition after 23 h	Mulholland et al., 2001
*Trichodesmium* spp. natural populations	Field manipulations	Glutamate	5 μM	Ambient surface seawater	23 h	33% inhibition	Mulholland et al., 2001
*Trichodesmium* spp. natural populations	Field manipulations	Glutamate	10 μM	Ambient surface seawater	0–23 h	5% inhibition after 1–2 h, 99% inhibition after 23 h	Mulholland et al., 2001
*Trichodesmium* spp. natural populations	Field manipulations	Glutamine	5 μM	Ambient surface seawater	23 h	89% inhibition	Mulholland et al., 2001
*Trichodesmium* spp. natural populations	Field manipulations	Glutamine	10 μM	Ambient surface seawater	0–23 h	29% inhibition after 1–2 h, 99% inhibition after 23 h	Mulholland et al., 2001
*Trichodesmium* sp. NIBB 1067	Batch culture	NO^−^_3_	1μM	3.2 μM	1–6 h	0% inhibition	Mulholland et al., 2001
*Trichodesmium* sp. NIBB 1067	Batch culture	NO^−^_3_	10 μM	3.2 μM	1–6 h	40% inhibition	Mulholland et al., 2001
*Trichodesmium* sp. NIBB 1067	Batch culture	NH^+^_4_	1μM	3.2 μM	2 and 4 h	0% inhibition	Mulholland et al., 2001
*Trichodesmium* sp. NIBB 1067	Batch culture	NH^+^_4_	10 μM	3.2 μM	2 and 4 h	90–99% inhibition after 4 h	Mulholland et al., 2001
*Trichodesmium* sp. NIBB 1067	Batch culture	Glutamate	1 or 10 μM	3.2 μM	2 and 4 h	0% inhibition after 4 h	Mulholland et al., 2001
*Trichodesmium* sp. NIBB 1067	Batch culture	Glutamine	1 or 10 μM	3.2 μM	2 and 4 h	Up to 50% inhibition after 2 and 4 h	Mulholland et al., 2001
*Trichodesmium* sp. GBRTRLI101	Batch culture	NH^+^_4_	2 μM	3 μM	3 generations	0% inhibition	Fu and Bell, 2003
*Trichodesmium* sp. GBRTRLI101	Batch culture	NH^+^_4_	10 μM	3 μM	1 generation	0% inhibition	Fu and Bell, 2003
*Trichodesmium* sp. GBRTRLI101	Batch culture	NH^+^_4_	10 μM	3 μM	5 generations	86% inhibition	Fu and Bell, 2003
*Trichodesmium* sp. GBRTRLI101	Batch culture	NO^−^_3_	10 μM	3 μM	1 generation	0% inhibition	Fu and Bell, 2003
*Trichodesmium* sp. GBRTRLI101	Batch culture	NO^−^_3_	10 μM	3 μM	5 generations	75% inhibition	Fu and Bell, 2003
*Trichodesmium* sp. GBRTRLI101	Batch culture	Urea	10 μM	3 μM	1 generation	0% inhibition	Fu and Bell, 2003
*Trichodesmium* sp. GBRTRLI101	Batch culture	Urea	10 μM	3 μM	5 generations	66% inhibition	Fu and Bell, 2003
*Trichodesmium* sp. IMS101	Continuous culture	NO^−^_3_	0.5–20 μM	10 μM	0–12 h (added just prior to initiation of light cycle)	Up to 35% inhibition up to 5 μM, ≥10 μM apparently saturates at 70% inhibition	Holl and Montoya, 2005
*Trichodesmium* sp. IMS101	Batch culture	NO^−^_3_	100 μM	50 μM	2 weeks	100% inhibition	Milligan et al., 2007
*Trichodesmium* sp. IMS101	Batch culture	NO^−^_3_	100 μM (semi-continuous re-supply of 100 μM NO^−^_3_)	50 μM	1, 3, or 6 days	~60% inhibition at 1 day, 100% inhibition at 3 and 6 days	Sandh et al., 2011
*Crocosphaera* sp. WH8501	Batch culture	NO^−^_3_	0.2–10 μM	50 μM	90 min prior to initiation of dark period	5% inhibition up to 1μM, 24% inhibition at 5 μM, 12% inhibition at 10 μM	Dekaezemacker and Bonnet, 2011
*Crocosphaera* sp. WH0003	Batch culture	NO^−^_3_	0.2–10 μM	50 μM	90 min prior to initiation of dark period	14% inhibition at 0.2 μM; 11% inhibition at 1μM, 4% inhibition at 5 μM	Dekaezemacker and Bonnet, 2011
*Crocosphaera* sp. WH8501	Batch culture	NH^+^_4_	0.2–10 μM	50 μM	90 min prior to initiation of dark period	Up to 12% inhibition up to 5 μM; 38% inhibition at 10 μM	Dekaezemacker and Bonnet, 2011
*Crocosphaera* sp. WH0003	Batch culture	NH^+^_4_	0.2–10 μM	50 μM	90 min prior to initiation of dark period	21% inhibition at 1μM, 41% inhibition at 5 μM, and 80% inhibition at 10 μM	Dekaezemacker and Bonnet, 2011
*Trichodesmium* sp. IMS101	Batch culture	NO^−^_3_	8 μM	0.5 μM	≥10 generations	90% inhibition^*^	Knapp et al., 2012
*Trichodesmium* sp. IMS101	Batch culture	NO^−^_3_	5 and 16 μM	1μM	≥10 generations	72% inhibition at 5 μM and 85% inhibition at 16 μM^*^	Knapp et al., 2012
*Crocosphaera* sp. WH8501	Batch culture	NO^−^_3_	8 μM	0.5 μM	≥10 generations	79% inhibition^*^	Knapp et al., 2012
*Crocosphaera* sp. WH8501	Batch culture	NO^−^_3_	5 and 16 μM	1μM	≥10 generations	71% inhibition at 5 μM and 85% inhibition at 16 μM^*^	Knapp et al., 2012

* Indicates the degree of inhibition when N_2_ fixation rates are normalized per trichomes or cells; N_2_ fixation is significantly less inhibited when N_2_ fixation rates are normalized to chl a content for Trichodesmium sp., but not for Crocosphaera sp.

The relatively small impact on N_2_ fixation rates by short-term exposure to NO^−^_3_ (Table 1) has implications for proposed mechanisms for diazotrophs to acquire limiting nutrients such as P. For example, short-term exposure to DIN could take place during the vertical migration of *Trichodesmium* spp. (Capone et al., 1990) showed that nitrogenase in *Trichodesmium* spp. is synthesized each morning prior to the initiation of nitrogenase activity. Consequently, the downward migration of *Trichodesmium* spp. at night (Villareal and Carpenter, 1990) to acquire P (Villareal and Carpenter, 2003) at the top of the nutricline (where NO^−^_3_ is also present) might not strongly depress peak daytime N_2_ fixation rates in *Trichodesmium* spp. if exposure to NO^−^_3_ is brief and occurs at night before new nitrogenase is synthesized. While studies of the effects of DIN inhibition on marine diazotrophs have largely been restricted to *Trichodesmium* spp., recent culturing work suggests that *Crocosphaera* has similar sensitivities to short-term vs. chronic NO^−^_3_ exposure (Dekaezemacker and Bonnet, 2011; Knapp et al., 2012). Given the similarity in response of *Trichodesmium* and *Crocosphaera* spp. and the limited genetic divergence in nitrogenase amino acid sequences in marine diazotrophic cyanobacteria (Zehr, 2011), the smaller effect of short-term vs. long-term DIN exposure on N_2_ fixation rates may be common among other diazotrophic cyanobacteria as well.

Culturing studies clearly show that DIN *can* inhibit N_2_ fixation; however most inhibition studies have been performed with concentrations of N and/or P in the culture media that exceed those typically found in the euphotic zone (Table 1). This discrepancy between nutrient concentrations in the environment and in cultures leaves open the possibility that culturing studies overestimate the degree to which DIN inhibits N_2_ fixation in the environment. Recent culturing work using concentrations of NO^−^_3_ and PO^3−^_4_ typically found in the euphotic zone show that chronic exposure of *Trichodesmium* and *Crocosphaera* to 5 to 16 μM NO^−^_3_ depresses N_2_ fixation rates relative to cultures grown with no NO^−^_3_, but that N_2_ fixation did not stop even in cultures amended with as much as 16 μM NO^−^_3_ (Knapp et al., 2012). Moreover, the same work showed that higher concentrations of PO^3−^_4_ can offset NO^−^_3_ inhibition of per-cell N_2_ fixation rates by increasing diazotroph abundance. Consequently, the volume-integrated rate of N_2_ fixation in treatments grown with 5.0 μM NO^−^_3_ and 1.0 μM PO^3−^_4_ was comparable to the volume-integrated rate of N_2_ fixation in treatments not amended with NO^−^_3_ and grown with 0.5 μM PO^3−^_4_ (Knapp et al., 2012).

The finding of increased diazotroph abundance as a function of increasing P availability is consistent with the well-recognized role that P availability plays in regulating the biomass of microbes [e.g., (Elser et al., 2007; Loladze and Elser, 2011; Scott et al., 2012)]. Investigations into variability in phytoplankton biomass N:P ratios indicate that P is preferentially used to create new biomass (e.g., in DNA) whereas N is required both for the production of new biomass as well as for the production of proteins, especially associated with resource acquisition (Klausmeier et al., 2004; Loladze and Elser, 2011). Consequently, the results of (Knapp et al., 2012) documenting a two- to three-fold greater abundance of both the diazotrophs *Crocosphaera watsonii* and *Trichodesmium erythraeum* in batch cultures grown with 1.0 vs. 0.5 μM PO^3−^_4_ are perhaps unsurprising. What is surprising is that the increase in diazotrophic biomass was sufficient to offset the lower per-trichome rates of N_2_ fixation resulting from inhibition by 5.0 μM NO^−^_3_. This work shows that NO^−^_3_ present at typical surface ocean concentrations does not necessarily preclude N_2_ fixation fluxes comparable to those observed in NO^−^_3_-depleted environments, and suggests that field and numerical modeling investigations of marine N_2_ fixation that exclude surface ocean environments with ≥1μM NO^−^_3_ may overlook potentially significant regions of N_2_ fixation.

Additionally, the work of (Knapp et al., 2012) identifies a potential mechanism for euphotic zone diazotrophs to respond to changes in surface ocean concentrations of NO^−^_3_ and PO^3−^_4_. Specifically, while it has been assumed that low ambient N:P ratios (a condition created by denitrification occurring below the euphotic zone) would stimulate higher rates of N_2_ fixation (Haug et al., 1998; Deutsch et al., 2004), no mechanism has been proposed for how a diazotroph would sense and respond favorably to lower N:P ratios. The results of Knapp et al. (2012) describe how the separate physiological effects of relatively high concentrations of P (i.e., increased diazotroph abundance) and relatively low concentrations of N (i.e., lessened NO^−^_3_ inhibition of N_2_ fixation) together can create conditions that can support significant N_2_ fixation fluxes. While relatively low N and high P concentrations have distinct effects on diazotrophs, combining these effects results in a perceived advantage for diazotrophs growing in environments with low ambient N:P ratios and may provide a feedback mechanism for diazotrophs to respond to increases in denitrification and thus help stabilize the marine N inventory. This finding also has implications for diazotroph biogeography, and suggests that significant abundances and/or N_2_ fixation fluxes may not be restricted to oligotrophic surface waters such as the North Atlantic, but may occur in more nutrient-replete regions of the surface ocean such as the surface waters overlying ODZs where rates of N loss are high.

Indeed, these culture-based results are consistent with recent field observations by (Fernandez et al., 2011; Sohm et al., 2011) who document N_2_ fixation rates of 0.1–7.5 nmol N L^−1^ d^−1^ in surface ocean waters with 5–20 μM NO^−^_3_ (Table 2), although molecular analyses indicate that this fixation was carried out by diazotrophs other than *Trichodesmium* or *Crocospahera* spp. These rates of N_2_ fixation in NO^−^_3_-replete coastal waters are comparable to the range in N_2_ fixation rates measured at Station ALOHA in the North Pacific gyre of 0.5–11 nmol N L^−1^ d^−1^ (Church et al., 2009) and where surface NO^−^_3_ concentrations are consistently ≤100 nM (Fujieki et al., 2011). Similarly, (Halm et al., 2012) found higher euphotic zone rates of N_2_ fixation in regions of the South Pacific gyre with higher concentrations of NO^−^_3_ (as well as PO^3−^_4_) compared to more oligotrophic regions of the gyre, i.e., average N_2_ fixation rates of 1.5 ± 0.3 nmol N L^−1^ d^−1^ vs. 0.4 ± 0.3 nmol N L^−1^ d^−1^, respectively. These N_2_ fixation rate measurements are supported by numerous other field observations documenting significant abundances of and/or N_2_ fixation by diazotrophs including *Trichodesmium* spp. in other near-shore locations (Lenes et al., 2001; White et al., 2007; Rodier and Le Borgne, 2008; Grosse et al., 2010; Rodier and Le Borgne, 2010; Bombar et al., 2011) (Table 2).

**Table 2:** **Reported rates of N_2_ fixation in euphotic, mesopelagic, and benthic marine environments with significant (i.e., ≥1μM) ambient concentrations of NO^−^_3_ and/or NH^+^_4_**.

**Location**	**Depth**	**Diazotroph**	**N_2_ fixation rate**	**N_2_ fixation method**	**Ambient [NO^−^_3_]**	**Ambient [PO^3−^_4_]**	**References**
**EUPHOTIC ZONE**							
Eastern Tropical North Atlantic	48 m	Unidentified, whole water incubation	0.7 nmol N L^−1^ h^−1^	^15^N_2_ assimilation	10 μM	0.6 μM	Voss et al., 2004
Mekong River plume, mesohaline station	Surface^*^	*Trichodesmium* spp.	1.13 nmol N L^−1^ h^−1^	^15^N_2_ assimilation	12.4 μM	0.7 μM	Grosse et al., 2010; Bombar et al., 2011
Benguela upwelling	8 m	Unidentified, whole water incubation	7.5 nmol N L^−1^ d^−1^	^15^N_2_ assimilation	21 μM	1.5 μM	Sohm et al., 2011
Eastern Tropical South Pacific	Surface^*^	Whole water incubation, 2005	0.089 nmol N L^−1^ d^−1^	^15^N_2_ assimilation	7.8 μM	1.2 μM	Fernandez et al., 2011
Eastern Tropical South Pacific	Surface^*^	Whole water incubation, 2007	0.66 nmol N L^−1^ d^−1^	^15^N_2_ assimilation	5.5 μM	0.68 μM	Fernandez et al., 2011
**MESOPELAGIC**							
Eastern Tropical South Pacific	400 m	Cluster I and III phylotypes	1.27 nmol N L^−1^ d^−1^	^15^N_2_ assimilation	>9.3 μM	>1.0 μM	Fernandez et al., 2011
California Borderland Basins	500 m	Heterotrophic Alpha- and Gammaproteobacteria, putative sulfate reducing bacteria	0.07 μmol m^−3^ d^−1^ total; <10 μm fraction 0.1μMol m^−3^ d^−1^, >10 μm fraction 0.01μMol m^−3^ d^−1^	^15^N_2_ assimilation	32 μM	4 μM	Hamersley et al., 2011
California Borderland Basins	850 m	Heterotrophic Alpha- and Gammaproteobacteria, putative sulfate reducing bacteria	0.07 μmol m^−3^ d^−1^ total; <10 μm fraction 0.08 μmol m^−3^ d^−1^; >10 μm fraction 0.00 μmol m^−3^ d^−1^	^15^N_2_ assimilation	32 μM	4 μM	Hamersley et al., 2011

**Location**	**Sediment depth**	**Diazotroph**	**N_2_ fixation rate**	**N_2_ fixation method**	**Ambient [NO^−^_3_]**	**Ambient [NH^+^_4_]**	**Ambient [PO^3−^_4_]**	**References**
**BENTHIC**							
Waccasassa estuary, FL, USA	Upper 2–5 cm	*Clostridium* spp.	0.64 – 6.0 ng N g^−1^ hr^−1^	Acetylene reduction	NR	0.06 mg N g–1	NR	Brooks et al., 1971
Continental Shelf Sediments, Upper Cook Inlet, AK, USA	Upper 0–5 cm	NR	0.3 μg atoms N m^−2^ hr^−1^^*^^#^	Acetylene reduction	29 μM	127 μM	NR	Haines et al., 1981
Continental Shelf Sediments, Norton Sound, AK, USA	Upper 0–5 cm	NR	0.8 μg atoms N m^−2^ hr^−1^^*^^#^	Acetylene reduction	7 μM	177 μM	NR	Haines et al., 1981
Zostera marina seagrass sediments, Long Island, NY, USA	Upper 12 cm	NR	1.6 nmol C_2_H_2_ cm^−2^ hr^−1^	Acetylene reduction	NR	116 μM	NR	Capone, 1982
Tomales Bay, CA, USA	Upper 1 cm	*Microcoleous* sp., *Lyngbya* sp., *Oscillatoria* sp., *Spirulina* sp.	3 mmol N m^−2^ d^−1^^*^^#^	Acetylene reduction	1μM	3 μM	1.9 μM	Joye and Paerl, 1993
Transplanted Spartina marsh, NC, USA	Upper 1 cm	Heterocystous and non-heterocystous cyanobacteria	37 mg N m^−2^ d^−1^^#^	Acetylene reduction	NR	18–83 μM	NR	Currin et al., 1996
Seagrass meadow, France	Upper 5 cm	Sulfate reducing bacteria	0.1–7.3 mg N m^−2^ d^−1^	Acetylene reduction	NR	190 μM	NR	Welsh et al., 1996
Mangrove sediments, Twin Cays, Belize	Upper 1 cm	Heterocystous and not-heterocystous cyanobacteria, sulfate reducing bacteria	0–1.21 mmol N d^−1^^#^	Acetylene reduction	1.0 μM	12–250 μM	0.4–1.9 μM	Lee and Joye, 2006
Corpus Christi Bay, TX, USA	Upper 15–20 cm	NR	0–75 μmol N m^−2^ hr^−1^^#^	Net N_2_ fluxes using MIMS	≤1μM	≤1μM	0.2–1.6 μM	McCarthy et al., 2008
Catalina Harbor sediments, CA, USA	Upper 0–10 cm	Sulfate reducing bacteria, cyanobacteria	0.1–8.0 mmol N m^−2^ d^−1^	Acetylene reduction	NR	50–100 μM	NR	Bertics et al., 2010
Eutrophic estuary, Waquoit Bay, MA, USA	Upper 20 cm	NR	0–0.77 mmol N m^−2^ hr^−1^^#^	Net N_2_ fluxes using benthic flux chamber and MIMS	<1μM	10–40 μM	NR	Rao and Charette, 2012

The units for N_2_ fixation rates as well as concentration are taken from the original publication.

* Indicates average value for N_2_ fixation rate, NO^−^_3_ concentration, and/or PO^3−^_4_ concentration;

# Indicates that denitrification was documented simultaneously in the same sediments; NR indicates not reported.

These reports of substantial rates of N_2_ fixation in NO^−^_3_-bearing surface waters, especially in upwelling and coastal regions, underscore the potential bias of prior field campaigns documenting N_2_ fixation predominantly in the nutrient-depleted oligotrophic gyres, and suggest that N_2_ fixation may have a broader geographic distribution in marine euphotic waters that episodically and/or chronically have significant DIN concentrations. Indeed, the strains of *Trichodesmium erythraeum* commonly used in culture studies, i.e., NIBB1067 and IMS101, were collected from the coastal waters of Japan and North Carolina, respectively (Ohki and Fujita, 1982; Prufert-Bebout et al., 1993), where surface water DIN concentrations are at least episodically elevated. That *Trichodesmium* spp. are frequently found in coastal waters that can have relatively high DIN concentrations is relevant considering that recent remote sensing (Westberry and Siegel, 2006) and geochemical modeling (Deutsch et al., 2007) studies have predicted high abundances of diazotrophs and/or rates of N_2_ fixation in regions of the surface ocean with NO^−^_3_ concentrations consistently ≥5 μM (Garcia et al., 2010). The results reviewed here suggest that NO^−^_3_ is not as inhibitive of N_2_ fixation by euphotic-zone diazotrophs as previously thought, especially if P and the necessary trace metals are abundant, and have implications for field studies documenting marine N_2_ fixation fluxes as well as for the parameterization of N_2_ fixation in models.

## Nutrient inhibition of mesopelagic N_2_ fixation

While there are only a handful of reports of N_2_ fixation occurring in the mesopelagic (i.e., sub-euphotic) water column, advances in molecular techniques capable of identifying diazotrophs and the improved sensitivity of mass spectrometers for detecting the incorporation of labeled ^15^N_2_ into suspended particulate organic N (PN_susp_) have improved our ability to evaluate N_2_ fixation in this environment. It is expected that N_2_ fixation in this portion of the water column would be carried out by diazotrophs that have substantially different physiologies than those living in the euphotic zone: mesopelagic diazotrophs require a different energy source than their photosynthetic counterparts, they need to tolerate lower temperatures, and due to the higher concentrations of NO^−^_3_ below the base of the euphotic zone, they would also presumably be less inhibited by NO^−^_3_. Perhaps unsurprisingly then, diazotrophs collected from meso- and bathypelagic waters contain *nifH* sequences distinct from euphotic zone diazotrophs. In samples collected from the deep North Pacific (Mehta et al., 2003, 2005) identified a number of *nifH* sequences associated with methanogens and anaerobic sulfate reducers from hydrothermal vent systems, and was able to document growth and N_2_ fixation in a culture of thermophilic archeal methanogens (Mehta and Baross, 2006). (Hewson et al., 2007) identified *nifH* genes in samples collected throughout the water column of the Sargasso Sea and detected *nifH* in meso- and abyssopelagic samples more consistently than in euphotic zone samples, suggesting the potential for diazotrophy below the euphotic zone. However, (Hewson et al., 2007) recovered *nifH* sequences of the cyanobacterial diazotrophs *Trichodesmium thiebautii* and *Crocosphaera watsonii* at 250 and 1000 m, respectively, demonstrating that the *nifH* associated with diazotrophs active in other environments persists upon transport to the deep ocean in a reasonably robust form, as has been recently reported for RuBisCO (Orellana and Hansell, 2012). However, (Hewson et al., 2007) also detected *nifH* expression in some mesopelagic samples, indicating some diazotrophs may be active in this NO^−^_3_-rich environment. Similarly, (Jayakumar et al., 2012) found both *nifH* DNA and cDNA sequences associated with strictly anaerobic proteobacteria in samples collected from the oxygen minimum zone of the Arabian Sea, also indicating potential activity of diazotrophs in sub-euphotic zone waters.

In addition to the molecular studies described above, two recent reports document relatively low rates of N_2_ fixation in mesopelagic samples collected from coastal environments. In the NO^−^_3_-rich coastal waters of the Eastern Tropical South Pacific (ETSP), (Fernandez et al., 2011) measured N_2_ fixation both in the euphotic zone and in mesopelagic waters, including in the core of the local oxygen deficient zone (ODZ) where they reported rates of 1.3 nmol N L^−1^ d^−1^ (Table 2). While (Fernandez et al., 2011) recovered numerous *nifH* sequences, they amplified no cyanobacterial phylotypes in surface or subsurface waters; instead most of the *nifH* sequences aligned with Cluster I, and to a lesser extent, Cluster III *nifH* genes, including representatives of anaerobic sulfate reducers. In mesopelagic samples collected in the California Borderland Basins (i.e., San Pedro and Santa Monica Basins) (Hamersley et al., 2011) report similar N_2_ fixation rates of 0.07 μmol N m^−3^ d^−1^ (Table 2). The most common *nifH* phylotype recovered by (Hamersley et al., 2011) was from the UCYN-A group found both in surface and mesopelagic samples. Additionally, (Hamersley et al., 2011) recovered heterotrophic *nifH* sequences in mesopelagic samples from Cluster I as well as a number of Cluster III sequences that correspond to strict anaerobes, including alpha- and gamma-proteobacteria, as well as sulfate reducing bacteria (SRB). While both (Fernandez et al., 2011; Hamersley et al., 2011) suggest that diazotrophy in these mesopelagic environments may be associated with oxygen deficiency, the similarity of some mesopelagic *nifH* sequences to those of diazotrophs found both in surface waters and in benthic environments (see below) raises the possibility that some of the diazotrophs recovered in these near-shore mesopelagic samples are introduced via sinking particles (from the euphotic zone) or via nepheloid layer from sediments to the water column further offshore. Given that a number of the phylotypes collected by (Hamersley et al., 2011) are similar to sequences from microbial mats and/or to cultivated strains of strict anaerobes, a condition not met in the water column of the San Pedro Basin where ambient oxygen concentrations are ~11μM, it raises the possibility that sedimentary microbes are resuspended and then detected in mesopelagic waters.

The determination of N_2_ fixation rates in mesopelagic waters presents unique analytical challenges as it depends on the incorporation of ^15^N_2_ by living, active diazotrophs into particulate organic matter that can then be analyzed by combustion on an isotope ratio mass spectrometer (Montoya et al., 1996). Even with increasingly sensitive instrumentation, the concentration of PN_susp_ in mesopelagic waters is extremely low. Thus, even with “large volume,” i.e., 4 L, incubations and given a typical detection limit of ~1.4 μmol N for GC-MS systems commonly used to analyze these samples (e.g., http://stableisotopefacility.ucdavis.edu/), a PN_susp_ concentration of ~0.35 μM is required to generate a signal above typical analytical detection limits. Since most open-ocean PN_susp_ concentrations are only this high within the euphotic zone, and then decrease sharply in the mesopelagic (i.e., PN_susp_ concentrations at 300 m at BATS and HOT are 0.05 μM) (Michaels and Knap, 1996; Fujieki et al., 2011), even larger volume incubations and/or more sensitive analytical approaches are required to reliably to detect N_2_ fixation rates in these waters. While PN_susp_ concentrations in mesopelagic waters of near-shore environments are higher than those in the oligotrophic ocean, e.g., (Hamersley et al., 2011) report PN_susp_ of 0.23 and 0.25 μM for their samples collected at 500 and 850 m, respectively, ensuring that mesopelagic samples have sufficient PN_susp_ to generate a signal above detection limits remains a significant challenge for documenting mesopelagic N_2_ fixation rates. Moreover, it is not clear that improving incubation techniques to increase ^15^N_2_ gas solubility (Mohr et al., 2010) will improve the ability to measure mesopelagic N_2_ fixation rates, as this modification does not increase the initial quantity of PN_susp_ in a mesopelagic sample. Given the very low PN_susp_ concentration in mesopelagic waters, great care must be taken to quantify blanks for these incubations and to demonstrate that N_2_ fixation rates generated by these methods contain a sufficient quantity of N to exceed analytical detection limits. Consequently, it may be warranted to view the water-column integrated mesopelagic N_2_ fixation rates of 55 μmol N m^−2^ d^−1^ in the California Borderland Basins (Hamersley et al., 2011) and 5.4 ± 2.4 μmol N m^−2^ d^−1^ in the ETSP (Fernandez et al., 2011), and their potential to help resolve global marine N budget imbalances, as provisional estimates until supporting measurements confirm the activity of N_2_ fixation in mesopelagic environments. If these early reports of relatively low N_2_ fixation rates in sub-euphotic zone waters (Table 2) are broadly characteristic of mesopelagic environments, they may be the consequence of NO^−^_3_ inhibition. A better understanding of the capacity of mesopelagic environments to support diazotrophy will benefit from methodological and analytical improvements of *in situ* N_2_ fixation rate measurements, as well as successful culturing of microbes recovered from these environments.

## Nutrient inhibition of benthic marine N_2_ fixation

From intertidal cyanobacterial mats to dark muds, and from low to high latitudes, numerous reports from diverse marine ecosystems demonstrate that benthic diazotrophy is widespread (Capone, 1983 and references therein). N_2_ fixation in marine sediments has received renewed attention based on evidence that the net flux of N_2_ gas in certain coastal sediments may have changed from efflux, via denitrification, to influx, via N_2_ fixation, potentially forced by climate change (Fulweiler et al., 2007). Due to the high concentrations of NO^−^_3_ and/or NH^+^_4_ that can accumulate as a result of organic matter degradation, N_2_ fixation in benthic environments presents perhaps the greatest challenge to the expectation for DIN to inhibit diazotrophy. Table 2 includes the small subset of all studies documenting benthic marine N_2_ fixation that reported both N_2_ fixation rates as well as concentrations of ambient NO^−^_3_ and/or NH^+^_4_ that exceeded 1μM. While the culture-based studies described above indicate that NH^+^_4_ significantly depresses N_2_ fixation rates in *Trichodesmium* and *Crocosphaera* spp., rates of 7–521μMol N m^−2^ d^−1^ have been documented in seagrass-bearing, NH^+^_4_-rich (190 μM) sediments on the French coast (Welsh et al., 1996). Similar rates have been reported in other NH^+^_4_-rich benthic environments, including mangrove sediments (Lee and Joye, 2006) and in coastal sediments from Alaska (Haines et al., 1981) to California (Bertics et al., 2010) to Florida (Brooks et al., 1971), indicating that benthic N_2_ fixation can occur at considerable rates in spite of high ambient NH^+^_4_ concentrations.

Given that the highest rates of N loss in the ocean occur in marine sediments (Brandes and Devol, 2002), it is perplexing that both N_2_ fixation and denitrification have frequently been observed in the same sediments (Haines et al., 1981; Slater and Capone, 1984; Joye and Paerl, 1993; Currin et al., 1996; An and Joye, 2001; Gardner et al., 2006; Lee and Joye, 2006; Fulweiler et al., 2007; McCarthy et al., 2008; Bertics et al., 2012; Rao and Charette, 2012). Indeed, *Azospirillum*, a bacteria associated with seagrasses (Patriquin, 1978) is thought to carry out both denitrification and N_2_ fixation (Bothe et al., 1981). These observations raise the question: if N_2_ fixation is an energetically costly process whose role is to provide a source of assimilatory N to the ecosystem, and if diazotrophs are inhibited by DIN, why does N_2_ fixation happen at significant rates in benthic environments rich in DIN and that also support denitrification?

Benthic diazotrophy has been investigated with a variety of biological and geochemical tools that together indicate that benthic N_2_ fixation is carried out by a diverse suite of microbes at environmentally significant rates (Table 2). Many benthic N_2_ fixation rates have been measured using acetylene reduction, and concerns have been raised regarding its use in these environments because of the capacity for acetylene to inhibit other microbial processes including denitrification, methanogenesis, methane oxidation, sulfate reduction, nitrification, and even N_2_ fixation [(Capone, 1983) and references therein]. In spite of these and other more general concerns regarding the limitation of methods to measure absolute rates of benthic microbial processes, including the high degree of spatial heterogeneity due to microsites and steep geochemical gradients on millimeter spatial scales, benthic diazotrophy has been validated using ^15^N_2_ assimilation (Patriquin and Knowles, 1972; Burris, 1976; Carpenter et al., 1978; Capone and Budin, 1982; Dekas et al., 2009) and net N_2_ gas flux measurements made using membrane inlet mass spectrometry (MIMS) (An and Joye, 2001; Gardner et al., 2006; Fulweiler et al., 2007; McCarthy et al., 2008; Rao and Charette, 2012). Based on visual identification of diazotrophs and differences in N_2_ fixation rates between light and dark incubations, cyanobacteria are thought to contribute to N_2_ fixation fluxes in intertidal microbial mat consortia (Joye and Paerl, 1993; Currin et al., 1996; An et al., 2001; Lee and Joye, 2006). Additionally, a number of benthic studies have used molybdate amendment experiments to inhibit sulfate reduction and have simultaneously inhibited N_2_ fixation in the same sediments; such experiments have been used to attribute N_2_ fixation in certain benthic marine environments to SRB (Gandy and Yoch, 1988; Welsh et al., 1996; Nielsen et al., 2001; Burns et al., 2002; Steppe and Paerl, 2002; Bertics et al., 2010). Molecular tools have also verified the presence of *nif* genes, and thus the metabolic potential for N_2_ fixation, in various benthic marine microbes including in SRB (Burns et al., 2002; Steppe and Paerl, 2002, 2005; Dekas et al., 2009; Bertics et al., 2010, 2012), anaerobic methane-oxidizing archaea (Dekas et al., 2009), and benthic cyanobacteria (Steppe and Paerl, 2005; Bertics et al., 2010).

Previous studies provide some insight into the role of DIN in regulating N_2_ fixation and denitrification in some benthic environments. Specifically, (Joye and Paerl, 1993, 1994) established seasonality in patterns of N_2_ fixation and denitrification in Tomales Bay, CA sediments that are consistent with studies documenting DIN inhibition of N_2_ fixation. (Joye and Paerl, 1993, 1994) observed that when ambient benthic DIN concentrations were relatively low, N_2_ fixation rates were high and denitrification rates were low, but when runoff or other sources introduced NO^−^_3_ to sediments, denitrification rates increased and N_2_ fixation rates decreased. These observations from Tomales Bay indicate both that denitrification is NO^−^_3_ limited and that N_2_ fixation is inhibited by NO^−^_3_. The sensitivity of benthic N_2_ fixation and denitrification rates to changes in ambient DIN concentration in Tomales Bay has been replicated in manipulated core studies and observed in other benthic N cycling studies. For example, in the estuarine sands of Waquoit Bay, MA (Rao and Charette, 2012) documented net N_2_ fixation, and suggested that denitrification occurring elsewhere in the estuary removes DIN, permitting N_2_ fixation to proceed downstream. Similarly, in a study of N_2_ fixation rates associated with seagrass roots in a French estuary (Welsh et al., 1996) observed peak N_2_ fixation rates when ambient NH^+^_4_ concentrations reached their annual minima of 190 μM, relative to the peak concentration of 290 μM.

Many of these studies also document complex interactions between oxygen, DIN, and/or organic carbon, and their relationship with N_2_ fixation and/or denitrification rates in benthic environments. For example, (Fulweiler et al., 2007) attributed a change from net denitrification to net N_2_ fixation in Narragansett Bay, RI sediments to a decrease in the organic matter flux to the sediments due to diminished winter-spring blooms in the Bay. (Fulweiler et al., 2007) tested this hypothesis, observing a change from net N_2_ fixation to net denitrification after adding organic matter to incubated sediment cores that had previously shown net N_2_ fixation. In the past, benthic remineralization of winter-spring bloom material in Narragansett Bay provided a source of DIN to the sediment and overlying water column, which is nutrient-poor in summers; presumably the reduction in the magnitude of the organic matter flux to Narragansett Bay sediments corresponds to a reduced DIN flux to the sediments, and is proposed by Fulweiler et al. (2007) to be the cause of the switch to net N_2_ fixation from net denitrification.

Observations of decreased rates of benthic N_2_ fixation when ambient DIN concentrations increase, either because of runoff or remineralization, are generally consistent with the observations described above that show that DIN inhibits, but does not stop, pelagic diazotrophy. However, the observations of decreased benthic N_2_ fixation rates when DIN concentrations increase are *not* consistent with other observations of high rates of benthic N_2_ fixation in dark, NH^+^_4_-rich environments [e.g., (Haines et al., 1981; Capone, 1982; Welsh et al., 1996; Bertics et al., 2010)]. Some previous studies of benthic N_2_ fixation have suggested that oxygen and organic carbon availability also play a role in mitigating DIN inhibition (Yoch and Whiting, 1986; McGlathery et al., 1998). Another explanation for why N_2_ fixation may occur at considerable rates in DIN-rich benthic environments invokes a role for N_2_ fixation that is entirely different from providing a source of assimilatory N to the ecosystem. Specifically, there is evidence that in the presence of high concentrations of NH^+^_4_ benthic N_2_ fixation can serve as a sink for excess electrons to help bacteria achieve redox balance, especially in the absence of a viable Calvin–Benson–Bassham pathway (Joshi and Tabita, 1996; Tichi and Tabita, 2000). Ultimately, sensitivity studies of benthic diazotrophs to these parameters are limited by the lack of isolated diazotrophs for manipulative culture studies.

A better understanding of the sensitivities of the diverse suite of benthic diazotrophs to oxygen, organic carbon and DIN is critical for refining models of benthic N cycling, and in particular determining whether marine sediments are a net source or sink of fixed N to the marine environment. While marine sediments are normally considered a net sink for fixed N (Seitzinger, 1988), a variety of reports show that some benthic environments can be a net source of bioavailable N at least on seasonal timescales, if not annually, as well (Currin et al., 1996; Lee and Joye, 2006; Fulweiler et al., 2007; McCarthy et al., 2008). Moreover, if environmental conditions change to favor diazotrophy (e.g., Fulweiler et al., 2007), it is plausible that even if marine sediments do not overwhelmingly become a source of fixed N, they might at least not be as large of a sink as previously thought. Benthic N_2_ fixation deserves more attention as it is a poorly constrained term in the global marine N budget; the process is not always included in marine N budget estimates, although (Capone, 1983) estimated it may contribute 15 Tg N year^−1^, which would increase some estimates of N fluxes to the marine environment by 10–15% (Brandes and Devol, 2002).

## Conclusions

Rates of the dominant fluxes of N to and from the ocean are highly uncertain, leaving open the question of whether the modern marine N budget is balanced. Some estimates suggest that rates of N fluxes to the ocean only compensate for one-third to one-half of the fluxes of N out of the ocean (Codispoti et al., 2001; Codispoti, 2007), while paleoceanographic and modeling studies require a balanced N budget, implying that either rates of N_2_ fixation are underestimated, and/or that rates of N loss are overestimated (Brandes and Devol, 2002; Deutsch et al., 2004). One potential liability in previous estimates of N fluxes to the ocean is the assumption that the highest rates of marine N_2_ fixation occur in the warm, nutrient-depleted regions of the surface ocean. However, culture and field evidence reviewed here indicates that low concentrations of NO^−^_3_ and/or NH^+^_4_ (≤1μM) are not a strict requirement for high rates of marine N_2_ fixation, and that numerical models using this as a criteria for significant diazotroph abundance and/or N_2_ fixation fluxes may not accurately represent diazotroph sensitivities to DIN. Generally, the best-studied cyanobacterial diazotrophs show little inhibition by short-term exposure to inorganic N. Instead, depressed rates of N_2_ fixation occur after long-term exposure of diazotrophs to elevated concentrations of DIN, although long-term exposure does not necessarily stop N_2_ fixation. Recent field and culturing work has shown that NO^−^_3_ concentrations commonly found in marine surface waters, i.e., up to 20 μM, do not preclude rates of N_2_ fixation comparable to those measured in the NO^−^_3_-depleted surface waters of the North Pacific gyre (Fernandez et al., 2011; Sohm et al., 2011). Moreover, field and culture evidence suggests that well-studied cyanobacterial diazotrophs such as *Trichodesmium* spp. are more tolerant of NO^−^_3_ than previously assumed, especially when P is relatively abundant. Together with molecular evidence documenting novel diazotrophs in cooler euphotic zone waters [e.g., (Needoba et al., 2007; Moisander et al., 2010)], these observations imply that surface waters other than those in the warm, nutrient-poor oligotrophic gyres may support substantial rates of N_2_ fixation, and that overlooking these potential diazotrophic contributions may compound uncertainties in the marine N budget, as well as modeled estimates of global marine diazotroph distributions and rates of N_2_ fixation.

While the nascent case for significant N_2_ fixation fluxes by mesopelagic diazotrophs is ambiguous, it is clear that N_2_ fixation occurs in diverse benthic environments at significant rates in the presence of DIN concentrations in excess of 100 μM. Benthic N_2_ fixation is peculiar in that it presents the strongest challenge to DIN inhibition of N_2_ fixation, and because it often occurs in environments that also support high rates of N loss via denitrification and/or anammox. While traditionally it has been thought that benthic environments represent a net loss of bioavailable N from the marine ecosystem, previous work has shown that the net flux of N_2_ gas to or from the sediments varies seasonally, and may be sensitive to environmental perturbations that may accelerate due to anthropogenic activities. These observations underscore the importance of developing and testing models of what controls benthic N_2_ fixation (and denitrification) to generate more robust estimates of benthic N fluxes.

Our current understanding of the sensitivity of even the most well studied marine diazotrophs is incomplete, and we have considerably more to learn about diazotrophs that have only recently been identified using molecular tools. These are critical uncertainties to resolve if we are to understand how the marine N inventory can remain balanced on 100–1000 year time scales. Better constraints of diazotroph sensitivities will help us understand N cycle changes in the past, and to predict future changes as atmospheric carbon dioxide concentrations and temperatures increase and potentially stimulate N_2_ fixation by *Trichodesmium* (Breitbarth et al., 2007; Hutchins et al., 2007; Levitan et al., 2007; Ramos et al., 2007; Levitan et al., 2010), if not other diazotrophs as well.

### Conflict of interest statement

The author declares that the research was conducted in the absence of any commercial or financial relationships that could be construed as a potential conflict of interest.
